# Spinal Irritation

**Published:** 1831

**Authors:** 


### LXIV.

### Spinal Irritation.

Mr. Rees, of Landilo, has published the following short case in our cotemporary, the London Medical and Surgical Journal.

William Jones, æt. two years and a half, a healthy, strong, and well-grown boy, had the misfortune of losing all command over his right lower extremity, in June, 1830. In the evening he was observed to be cheerful, and walking about in his usual manner, but when taken out of bed the following morning, he was discovered, to the no moderate grief of his parents, to be incapable of the slightest motion. In a few days after this happened, I was requested to attend him, and from my notes, I find that he was then very feverish, and that his stomach and bowels were in a very disordered state. I soon reinstated him in good general health, by the exhibition of purgative medicines; and as I then conceived the state of his limb to be, in a great degree, if not entirely, dependent on the disordered state of his digestive organs, I expected an improvement in the former, when the latter should be brought to execute their proper functions. But in this I was disappointed. The parents, not finding their boy's leg immediately beginning to improve, consulted (as is generally the case with the middle and lower class of this country) another surgeon, I therefore had no opportunity of trying other remedies, until I returned from the metropolis in June last, when I was entreated to attend the boy again. The following are the notes I then took:—Cannot move his right leg in the slightest degree; sensibility much diminished in this limb; its muscles are very flaccid; it is smaller than the left leg in circumference; its temperature is below par. The fulness of the glutæi muscles of the right side, much less than the glutæi of the left. He seems to experience no pain when his leg is moved about, or when extended, and the sole of the foot, strongly tapped with the hand. Bowels regular, and general health very good.

In examining the spine, I discovered some tenderness on pressure in the lower part of the lumbar region and upper part of the sacrum. I immediately applied a cupping-glass to the tender part of the back, and took away about an ounce and a half of blood. The following day he asked his mother to let him down, that he might walk; she indulged him in this, and to her great joy, he managed to limp along for some yards. Since then he has been again cupped, and counter-irritation has been kept up in the back for some weeks, by the ung. antim. tart. The boy was brought to me yesterday, when I had the gratification of seeing him walk about pretty well, but he is rather lame. He has for the last three weeks been affected with hooping cough, of no ordinary severity, and his mother, on this account, has suspended the application of the ointment. When he improves a little, I intend pursuing further the same course of treatment.

